# Cardiometabolic shock: understanding the final turns of the downward spiral

**DOI:** 10.3389/fcvm.2025.1653306

**Published:** 2025-09-29

**Authors:** Tobin Mathew, Jin Kyung Kim, Michael A. Gibson

**Affiliations:** ^1^ Division of Cardiology, Department of Medicine, University of California, Irvine, CA, United States; ^2^ Division of Cardiology, Department of Medicine, Sutter Medical Center, Sacramento, CA, United States

**Keywords:** cardiometabolic shock, cardiogenic shock, inflammation, lactic acidosis, mechanical circulatory support

## Abstract

Cardiogenic shock (CS) is a state of decreased cardiac output leading to systemic hemodynamic collapse and potential end-organ damage with an elevated risk of mortality. CS represents a heterogenous disease state with varying etiologies, severities, and hemodynamics. Several attempts have been made to characterize CS, including the Society of Cardiovascular Angiography & Interventions shock classification (SCAI), the American Heart Association (AHA) hemodynamic phenotypes, as well as other groups defining shock by underlying clinical factors and pathophysiology. Here, we review cardiometabolic shock, a complex and severe form of CS characterized by severe lactic acidosis and metabolic derangement, systemic inflammation with ischemia/reperfusion injury, persistent vasodilation despite hemodynamic support, and right heart failure, culminating in progressive end-organ failure and a downward spiral of cardiovascular instability. Understanding the components of pathophysiology underlying cardiometabolic shock may help to establish more accurate diagnosis and instituting prompt therapy in the management of this grave cardiac illness. The emerging roles of nitric oxide synthase inhibition, antioxidants, anti-inflammatory agents, proteomics, and artificial intelligence are discussed. Further studies are needed to fully understand cardiometabolic shock and to develop specific effective therapeutic targets.

## Introduction

Cardiogenic shock (CS) is a complex hemodynamic state of diminished cardiac output and end-organ hypoperfusion, as a result of an acute insult to the heart, such as myocardial infarction (MI), myocarditis, or decompensated heart failure, often associated with hypoxia (1, 2). CS is typically thought of as a mechanical failure manifesting as decreased contractility of the myocardium, leading to decreased cardiac output and subsequent end-organ malperfusion. This form of shock has been typically thought of as mechanistically distinct from other forms of shock, such as obstructive or distributive shock. One of the hallmarks of all shock states includes systemic ischemia which overwhelms tissue ability to compensate via metabolic and vascular modulations. Once these systems are overwhelmed, homeostatic capacity is overwhelmed and typically leads to a rapid decline in organ function and subsequent worsening shock (3). However, growing evidence suggests that there is a large metabolic and inflammatory component of CS, particularly as the disease progresses (2, 4). Classically CS is characterized as having a “cold and wet” presentation with peripheral vasoconstriction leading to increased systemic vascular resistance (SVR) (1). Although reflex vasoconstriction may temporarily improve coronary and systemic perfusion, the increased afterload eventually leads to further cardiac dysfunction through decreased cardiac output, decreased coronary perfusion and subsequent myocardial ischemia.

Despite the classical paradigm of depressed cardiac output leading to compensatory vasoconstriction with an elevated SVR, it is well established that the SVR may vary widely in patients with CS. Patients may exhibit a “warm and wet” profile with a paradoxically low or inappropriately normal SVR, which portends a greater mortality than CS with a high SVR (5). Hypotension and subsequent hypoperfusion may lead to significant lactic acidosis, which also has been associated with higher mortality in patients with CS (6, 7). These processes are a direct result from ischemia/reperfusion (I/R) injury from CS and are caused by the systemic oxidative stress and immune response (8). I/R occurs when cells undergo hypoxia which leads to impaired energy production, causing cell death and release of pro inflammatory mediators (9). Evidence suggests that the inappropriately low SVR in CS may be mediated by cytokine release such as interleukin (IL)-6, IL-8, tumor necrosis factor-α (TNF-α), C-reactive protein (CRP), and other vasodilatory mediators such as nitric oxide (NO) (10, 11). These molecules have further been shown to have prognostic value regarding mortality in patients with CS (2). The prominent features of severe acidosis and metabolic derangement, profound hypotension without the compensatory rise in SVR, and activation of the inflammatory signaling pathways found in cases of CS has led to the use of the terms “hemometabolic shock,” conveying the hemodynamic and metabolic components, and “cardiometabolic shock” signifying the cardiac etiology and subsequent metabolic consequences of shock. These two terms have been used interchangeably in current literature. For the remainder of the current document, we will be using the term “cardiometabolic shock” as this review will be limited to shock secondary to cardiac etiology. The cardiometabolic shock phenotype often represents a state of treatment-resistant shock, in which patients continue to deteriorate clinically despite mechanical and pharmacologic support. Cardiometabolic shock is a state that is currently poorly understood, as it has only recently been defined. Treatment options specific to this classification of shock remain unclear; however, early recognition and prevention of further deterioration of hemodynamic instability and end-organ dysfunction may lead to improved outcomes. The goal of this review is to further delve into cardiometabolic shock and discuss its unique pathophysiology and contributing characteristics through existing contemporary literature.

### What defines cardiometabolic shock?

**Figure 1:**
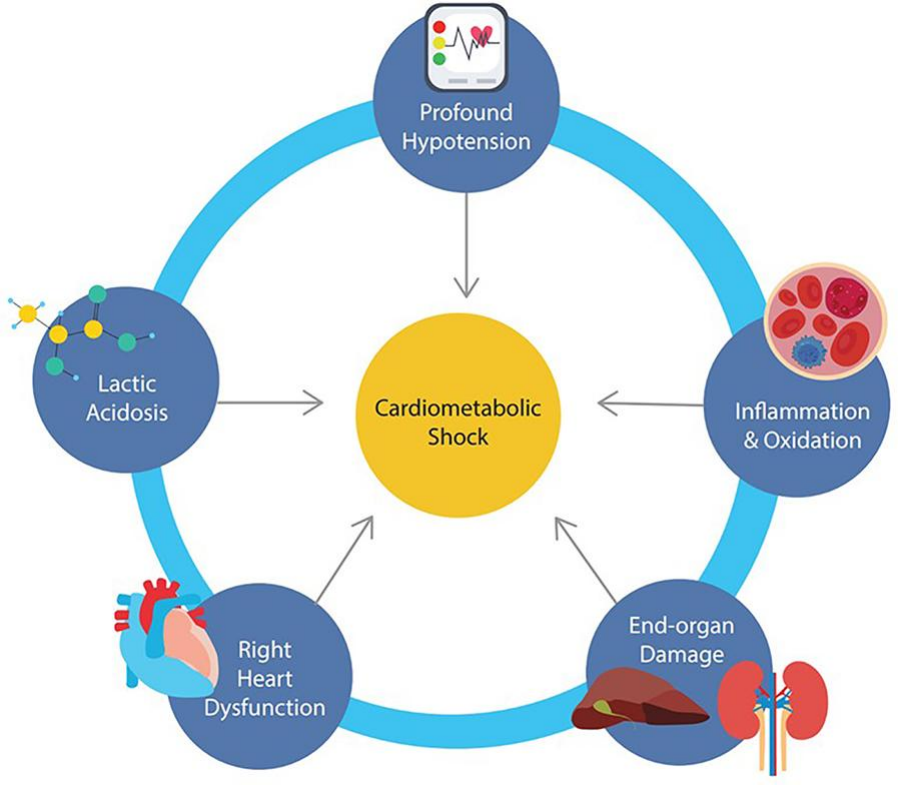
Schematic illustrating the interconnected processes driving cardiometabolic shock including profound hypotension, lactic acidosis, systemic inflammation, right ventricular dysfunction, and multiorgan injury.

There have been multiple trials and guidelines to define and classify CS (12). Recently, the Society of Cardiovascular Angiography & Interventions (SCAI) proposed a staging system of CS from A to E, classifying CS into progressive stages based on clinical and hemodynamic criteria (13). According to the SCAI staging, stage A is an at-risk group, stage B “beginning” shock, and stage C the classic CS, with hemodynamic instability defined as hypotension with tachycardia that requires pharmacologic or mechanical support to aid in end-organ perfusion. Stage D is deterioration from stage C, needing multiple inotropes, vasopressors, or addition of mechanical circulatory support devices, while stage E represents the patient being in extremis with unstable hemodynamic status and cardiovascular collapse despite all instituted therapies. The 2017 American Heart Association (AHA) Scientific Statement on Contemporary Management of Cardiogenic Shock describes 3 hemodynamic phenotypes of CS in terms of peripheral circulation and volume status (12). In the AHA statement, patients who are “cold and wet” with increased SVR and pulmonary capillary wedge pressure (PCWP) are denoted as classic CS. “Cold and dry” CS (increased SVR and normal PCWP) represents an euvolemic patient with subacute decompensation of chronic heart failure. “Warm and wet” CS with low to normal SVR and elevated PCWP is described as mixed or vasodilatory CS. “Warm and dry” is not considered cardiogenic shock. Cardiometabolic shock (Figure 1) is best represented by the AHA “warm and wet” vasodilatory CS given its association with systemic inflammatory response syndrome (SIRS), elevated nitric oxide synthase (NOS) expression, decreased SVR, and elevated mortality.

The CS subtypes were further stratified by Zweck et al. who used machine learning algorithms to identify and characterize clusters of patients within CS cohorts [Cardiogenic Shock Work Group Registry for both MI and heart failure (CSWG-MI and CSWG-HF respectively), and the Danish Retroshock MI Registry (DRR)] based on predictive variables. The authors described three CS phenotype clusters: non-congested CS, cardiorenal CS, and cardiometabolic shock (14). While the SCAI staging system reflects disease severity throughout the duration of a hospitalization, the phenotype clusters characterize patients based on demographic and clinical characteristics as well as metabolic and hemodynamic variables at the time of initial presentation. Non-congested CS represented a relatively stable profile with lower heart rate and cardiac filling pressures, and relatively higher blood pressure. Cardiorenal CS was more common in older patients with more comorbidities and higher pulmonary artery pressure and PCWP, as well as worse renal function secondary to shock. The cardiometabolic phenotype had substantially higher levels of lactate as well as end organ dysfunction and inflammation. These patients had much more profound shock with lower mean arterial pressures (MAP), lower cardiac index (CI), and higher heart rate, when compared to those with non-congested or cardiorenal CS. The cardiometabolic shock profile also had higher rates of right ventricular (RV) failure with higher right atrial pressures and lower pulmonary artery pulsatility indices (14). Cardiometabolic shock had an elevated PCWP similar to other phenotypes of cardiogenic CS. Findings by Jentzer *et al*. added to further characterization of cardiometabolic shock, confirming higher levels of lactate, acidemia, transaminitis, poor renal function, and neutrophil-predominant leukocytosis in those with cardiometabolic shock. Though mean left ventricular (LV) ejection fraction did not differ between these subgroups of shock, the cardiometabolic phenotype was found to have highest rates of moderate or severe RV dysfunction, highest right atrial pressure, and lowest stroke volume/index, cardiac output/index, cardiac power output, and LV stroke work index. Another recent study by Soussi et al. differentiated four biomarker-driven CS phenotypes from two prospective CS cohorts and found an independent association between endothelial dysfunction and inflammatory biomarkers with higher mortality (15). Comparison of the discussed CS phenotypes are summarized in Table 1.

**Table 1:** Clinical, hemodynamic, and metabolic characteristics of cardiogenic shock phenotypes.

	Classic/Vasoconstricted CS^a^	Non-congested/Euvolemic CS	Cardio-renal CS	Cardiometabolic/Vasodilatory CS
Profound hypotension and tachycardia	–	↔	↑	↑↑
Elevated lactic acid	–	↔/↑	↑	↑↑
Inflammation	–	↔	↔	↑↑
Right heart dysfunction	–	↔	↔	↑↑
End organ dysfunction	–	↔	↑↑	↑↑↑
Elevated pulmonary capillary wedge pressure	↑↑	↔	↑↑	↑
Systemic vascular resistance/mean arterial pressure	↑	↑	↔	↓↓
Cardiac output	↓	↓	↓	↓↓
Mortality (in hospital 30-day)	30–50%	10–30%	30–50%	50–60%

Comparison of American Heart Association (AHA) hemodynamic phenotypes and machine learning derived clusters described by Zweck et al. The cardiometabolic phenotype, corresponding to vasodilatory/mixed CS, demonstrates the most profound acidosis, systemic inflammation, right ventricular dysfunction, multiorgan injury, and the highest mortality when compared to other phenotypes.

^a^ The AHA definition of classic CS includes only hemodynamic parameters.

### Prognosis of cardiometabolic shock

Cardiometabolic shock is predominantly comprised of SCAI shock stages D and E, reflecting the disease severity of this phenotype in the spectrum of CS. In the retrospective study by Zweck, the cardiometabolic shock cluster exhibited a higher mortality rate than the other CS subtypes, with a mortality of 52%–56%, 10%–28% for non-congested CS, 32%–45% for cardiorenal CS, and 30%–50% for classic CS (12, 14, 16). The variation in mortality is accounted for by the range of mortalities for the respective CS subtypes in each of the used registries (CSWG-MI, CSWG-HF, and DRR) for the study. When stratified by each SCAI stage, the cardiometabolic shock phenotype had the highest mortality compared with the other phenotypes. This suggests that the increased mortality in cardiometabolic shock may be largely driven by the high prevalence of SCAI stage E among these cases. In addition, studies have consistently shown that the cardiometabolic shock phenotype has the highest mortality rates among all types of shock, cardiac or noncardiac (17–19). A study by Jentzer et al. demonstrated cardiometabolic shock to have an elevated one-year mortality compared to other forms of CS; with odds ratio 2.6 vs. non-congested CS and 2.0 vs. cardiorenal CS (20). The elevated risk of mortality may be attributed to the complex and systemic pathophysiology associated with cardiometabolic shock (6, 21).

### Pathophysiology of cardiometabolic shock

Distinguishing features of cardiometabolic shock is summarized in Figure 1 and include: (1) profound hemodynamic instability with persistent hypotension despite vasopressor support, (2) marked lactic acidosis and metabolic derangement, (3) evidence of systemic inflammation, (4) development of right heart dysfunction, and (5) end organ damage involving renal and hepatic injury. While one unifying mechanism behind the pathophysiology of cardiometabolic shock has not been identified, and the presence of one or more of these factors may trigger each other in a vicious cycle, it would be important to understand these abnormal components within the context of CS for better risk stratification and management.

### Profound hypotension and tachycardia

CS is a state of decreased cardiac output from an insult to the myocardium, leading to hypotension and hypoperfusion. In cardiometabolic shock, significant lactic acidosis and SIRS result in worsening vasodilation and decreased SVR, in contrast to compensatory vasoconstriction seen in the other CS subtypes (22). In classic CS, early I/R leads to the activation of the renin-angiogensin-aldosterone system (RAAS), which leads to vasoconstriction (9). However, unopposed systemic tissue damage from prolonged I/R injury can lead to inappropriate vasodilation (3). One of the mechanisms behind paradoxical vasodilation is thought to be due to the release of NO via inducible NOS (iNOS). Compared to other predominant constitutively expressed isoforms of NOS, neuronal NOS (nNOS) and endothelial NOS (eNOS), iNOS is induced by inflammation, infection, and endothelial damage. Another isoform of NOS and proposed subunit of nNOS, mitochondrial NOS (mtNOS), is constitutively present on mitochondrial matrix and inner membrane involved in oxidative phosphorylation. mtNOS been shown to also play a significant role in development of reactive oxygen species and apoptotic pathways under circumstances of physiological stress and inflammation (23, 24). Following iNOS induction, the supraphysiological amount of NO generated by iNOS mediates massive arteriolar vasodilation and hypotension, as seen in septic shock (25). In the context of coronary ischemia, it has been shown that the NO production by the infarcted heart accounted for the increase of NO concentration in circulation (26). eNOS, by contrast, is mainly expressed in endothelial cells and cardiomyocytes, and regulates physiological actions of NO in several key aspects of cardiovascular homeostasis (27). Normal NO production from eNOS causes vascular smooth muscle relaxation, reduces oxidative stress, and inhibits platelet aggregation in a manner that is cardioprotective (28, 29). However, in patients with refractory CS, elevated levels of NO generated by iNOS are thought to lead to inappropriate vasodilation and coronary hypoperfusion, further exacerbating hemodynamic instability and treatment resistance. Thus, inhibiting iNOS action has been a subject of interest among those involved in the care of patients with refractory CS (30, 31). In animal models, deleting iNOS genes improved coronary flow and survival after MI (30). NO-derived agents, such as peroxynitrites, were also shown to decrease myocardial contractility, increase inflammation, and induce further systemic vasodilation (29, 32). However, a randomized clinical trial testing the efficacy of NOS inhibition in patients with CS, the TRIUMPH (Tilarginine Acetate Injection in a Randomized International Study in Unstable Acute Myocardial Infarction Patients/Cardiogenic Shock) trial, demonstrated that tilarginine, a nonselective NOS inhibitor, did not alter mortality following MI (33). Whether the failure of the TRIUMPH trial to show benefit, in contrast to earlier smaller clinical studies with positive results, is due to the nonselective nature of NOS inhibition of tilarginine and concurrent suppression of cardioprotective eNOS activity, is unknown. The concept of phenotypes of CS with possible varying degrees of iNOS activity may also confound results as each phenotype may respond differently to these agents. Similarly, subgroup analysis of the stages of CS studies may provide further guidance as to the optimal timing and severity in which these agents are beneficial.

Higher heart rate is found in cardiometabolic shock, compared to other phenotypes of CS (14). All forms of shock lead to initial compensatory tachycardia via sympathetic activation to maintain cardiac output and SVR in the setting of global hypoperfusion (34). However, in cardiometabolic shock, tachycardia may be maladaptive, increasing myocardial demand and worsening the already poor cardiac function (5). While the exaggerated response of tachycardia may be in part due to the drop in stroke volume, significant metabolic and inflammatory components may contribute to the pronounced tachycardia and the worse hemodynamic profile of cardiometabolic shock (35).

### Lactic acidosis

Lactic acidosis has been identified as one of the defining features of cardiometabolic shock (14, 20, 21). Lactic acid is a well-established marker of hypoperfusion and tissue hypoxia, as its level rises with the extent of anaerobic metabolism in the setting of hypoxia or demand-ischemia and I/R, and its clearance hampered by poor perfusion (36). Metabolic acidosis, including lactic acidosis, has also been shown to decrease cardiac contractility and blunt vascular response to pharmacologic vasopressors (37). Acidosis itself has significant effects on action potential and excitation-contraction coupling of myocytes, including desensitization of the ryanodine receptor, decreased calcium release from the sarcoplasmic reticulum, and subsequent attenuation of myocyte contractility (38). In addition, an acidic extracellular pH has been shown to decrease myocardial *β*-adrenergic receptor expression, a form of G-protein coupled receptors (GPCR), resulting in a lack of response to both endogenous and exogenous catecholamine stimulation (39). Normal receptor function signaling requires these GPCRs to interact with identical or non-identical receptors (homodimerization and heterodimerization, respectively) (40, 41). Changes in pH significantly affect the ability for GPCRs to dimerize, which helps to explain in part why cardiometabolic shock is more resistant to vasopressor and inotropic support (42). Similarly, lactic acidosis disrupts the intracellular calcium homeostasis of vascular smooth muscle cells and internalization of adrenoreceptors from the cell surface. This, in turn, causes vascular smooth muscle cell relaxation and vasodilation. Furthermore, lactic acidosis induces the expression of iNOS in vascular smooth muscle cells, adding to the vasodilatory effect (37). An elevated level of intracellular lactic acid also triggers a mitochondrial release of pro-apoptotic factors, such as cytochrome *c*. Increased cardiac myocyte apoptosis may worsen the ventricular dysfunction at the organ level. Indeed, acidosis has been shown to impact RV dysfunction as well as electrophysiologic abnormalities (43, 44). Hepatic injury caused by cardiometabolic shock impairs lactate clearance which acts to further impair myocyte function and lead to worsening shock.

Among patients with CS, the level of acidosis is associated with severity of shock and increased mortality (14). Cardiometabolic shock is most strongly associated with elevated lactate than with other metabolic variables examined, including elevated transaminases, electrolyte abnormalities, abnormal bicarbonate, and hematologic markers. This contrasts with the other phenotypes of CS which lack a robust correlation with lactate. Lactic acidosis and severe acidosis have also been shown to be independent predictors of mortality in cardiometabolic shock with an overall mortality of 64.8% vs. 37.6% in patients without severe acidosis (6). An elevated admission lactate (>5 mmol/L) or acidemia (pH <7.2) were both independently associated with unadjusted in-hospital and 30-day mortality (21). Treatment specific to resolve severe lactic acidosis is limited. Current literature suggests the benefit of bicarbonate treatment of severe metabolic acidosis as defined as pH <7.1 or serum bicarbonate <6 mEq/L (45). Specifically, 30-day mortality of patients with severe acidosis with concomitant acute kidney injury (AKI) was improved with bicarbonate infusion, along with decreased rates of renal-replacement therapy and vasopressor use (63% *vs.* 46% survival). In addition to bicarbonate therapy, renal replacement therapy (e.g., continuous renal replacement therapy, hemodialysis) can help alleviate metabolic acidosis. However, there is not enough evidence to date to suggest renal replacement therapy improves CS or mortality in intensive care unit patients (46, 47).

### Systemic inflammatory processes

The landmark Should We Emergently Revascularize Occluded Coronaries for Cardiogenic Shock (SHOCK) trial investigated early revascularization *vs*. medical treatment in patients with LV failure following an acute MI and demonstrated a lower 6-month mortality rate in patients with early revascularization compared to those with early medical stabilization. One-fifth of the patients with CS in the SHOCK trial demonstrated clinical signs consistent with SIRS (4). In such patients, the median SVR was lower compared to patients without SIRS independent of vasopressor use. Many of these patients remained culture-negative and free of culpable infection to explain the inappropriate response to CS, as decreased cardiac output should lead to compensatory systemic vasoconstriction and elevated SVR (12). Individuals with CS and signs of severe inflammation were shown to have a higher mortality rate when compared to patients with CS without inflammation or culture-negative sepsis (4, 6). This suggests a separate and compounding pathophysiology when CS is associated with severe inflammation, with or without infection. During an acute MI, the normal immune response includes both a pro-inflammatory phase as well as a reparative phase with tissue remodeling. Normal physiology has natural regulatory processes to suppress inflammation, including by suppressing antigen presentation and T-cell deactivation in a process known as Compensatory Anti-Inflammatory Response Syndrome (CARS) (3). As this regulatory process interacts with SIRS, there is coexistence of both pro and anti-inflammatory states, which can lead to more immune dysregulation in a period also referred to as Mixed Antagonistic Response Syndrome (MARS). MARS can exist as an equilibrium between these two forces but often represents a tenuous metabolic state which has the tendency to deteriorate once the system surpasses its compensatory capacity. This leads to deleterious processes including worsening ischemia/reperfusion injury. In some circumstances, this can lead to increased susceptibility to infections despite a systemwide immune response, and also to vasodilation.

I/R injury cause cardiomyocyte death, leading to the release of intracellular contents and activating the innate immune system to promote inflammation via complement and damage-associated molecular patterns (DAMPs) (48, 49). Cardiac fibroblasts release an array of inflammatory cytokines and chemokines including IL-1 (*α* and *β*), IL-6, and TNF-α. IL-1 has been shown to be a major mediator of the inflammatory response (50). IL-1*α* is seen to predominate the acute pro-inflammatory phase, whereas IL-1β has been shown to reduce inflammation and subsequent infarct size. IL-6 has been shown to have both pro- and anti-inflammatory roles in acute MI and is a predominant circulating cytokine in patients with CS (20, 51, 52). It has early prognostic value to clinical outcome, including elevated mortality (11, 53). Patients with higher levels of IL-6 (>307 pg/ml) on admission are more refractory to mechanical circulatory support with worse clinical outcomes. Following implant of mechanical circulatory support, survivors of CS tended to have reduced IL-6 levels while subsequent levels in non-survivors continued to rise (54). Animal studies have shown that therapies antagonizing IL-6 actions reduced cardiac dysfunction by decreasing systemic inflammation (55). Overall, these data suggest IL-6 to be a promising potential therapeutic target in cardiometabolic shock in the setting of inappropriate systemic inflammation.

The proinflammatory state of cardiometabolic shock is regulated by multiple immune cell lines. Neutrophils as part of the innate immune system clear debris and dead tissue (56). There is a well-established post-injury neutrophil surge during the pro-inflammatory state after an acute MI; however, prolonged neutrophil activity has been shown to lead to poorer prognosis after MI in animal models (22). Neutrophil to lymphocyte ratio (NLR) is a marker for systemic inflammation, and higher NLRs have been shown to be associated with poorer survival in a variety of pro-inflammatory states (57, 58). NLRs >3.36 after undergoing coronary artery bypass grafting was strongly associated with higher mortality (57). Lower NLR, on the other hand, suggests a clinically favorable trajectory. It was associated with earlier stages of SCAI, and patients on mechanical assist device support who had lower NLRs had lower mortality rates (59, 60). Monocytes also play a key role in inflammation through their interactions with other cells via release of cytokines (e.g., IL-10, TNF-α) and reacting to inflammatory markers by differentiating into terminal macrophages (61). The release of pro-inflammatory cytokines such as TNF-α, IL-1, and IL-6 by neutrophils and monocytes have been shown to lead to the release of reactive nitrogen species, nitric oxide, and reactive oxygen species (ROS) during the process of phagocytosis (61, 62).

Another immune cell line relevant in the context of cardiometabolic shock is eosinophils whose functions involve tissue repair and remodeling (10). Eosinophils are recruited from the bone marrow and regulate the actions of IL-4 and IL-5, which help transition immune response to a reparative phase (63). Eosinophil levels increase in the serum post-MI and decrease after revascularization (10, 64). Severe eosinopenia following the initial spike in the serum level has been associated with higher rates of cardiac events and poor myocardial repair, while a delayed eosinophil surge has been suggested as a sign of dysregulated immune response associated with higher rates of re-infarction and death (64).

Regulatory T cells (Tregs) and differentiated macrophages are key to the resolution of inflammation (22). Recruited by anti-inflammatory mediators such as IL-10 and transforming growth factor beta (TGF-*β*), Tregs provide mechanisms for physiologic attempts of immune self-regulation and promote revascularization. Tregs have been shown to reduce myocyte apoptosis, contribute to cardiac regeneration in zebrafish, and facilitate cardiac repair by limiting negative remodeling in mice (65, 66). While limited clinical data exist on the role of Tregs in CS, a small observational study demonstrated that CS-non-survivors had the lowest levels of Treg cells and that the ratio between Treg and the pro-inflammatory T cell subset, helper T type 17 (Th17) cells, was prognostic of mortality in patients with CS (67).

In sum, experimental and human studies in aggregate suggest a strong line of evidence that the exaggerated or dysregulated inflammatory response plays a critical role in severe CS. While much research is needed to fully understand the role of inflammation in cardiometabolic shock, the data to date opens a promising possibility of novel treatments in the modern era of rapidly advancing immunotherapy.

### Reactive oxygen species

Inflammation and I/R injury contributes to the formation of ROS due to membrane destabilization (3). ROS have an important role in the cellular repair mechanisms, and participate in intracellular homeostasis and cell fate by triggering apoptosis in cells with significant damage (68). However, excessive oxidative stress from ROS generation can lead to severe and irreversible harm to cardiomyocytes. ROS production in cardiomyocyte mitochondria has been strongly associated with post-MI I/R injury and heart failure, with loss of viable cardiomyocytes via apoptosis, extracellular fibrosis, decreased myocyte contractility, and the eventual progression to heart failure (69, 70). Abnormalities in mitochondrial homeostasis have been implicated in various cardiac diseases, including ischemic heart disease (71). As lactate is actively oxidized in a hypoperfused state of cardiometabolic shock, high levels of ROS are produced in mitochondria, leading to oxidative damage (72). In CS patients, peak values of oxidized guanine species (OGS), a surrogate marker of ROS, were found within the first 24 h of CS. Non-survivors of CS were found to have OGS peak earlier and significantly higher levels of other surrogates of ROS, such as Cu/Zn-superoxide dismutase and total antioxidant capacity, compared to survivors (73). Inhibiting oxidative stress may result in improved outcomes in CS and a potential target for therapy in cardiometabolic shock. A pilot study by Guariento et al. evaluated autologous mitochondrial transplantation in pediatric patients with CS undergoing veno-arterial extracorporeal life support (VA ECLS) (74). Although a small study (24 patients), the authors demonstrated that cardiovascular events were lower in the mitochondrial transplantation group (20% vs. 79%; *P* < .01) in patients with severe refractory CS after ischemic reperfusion injury. Further studies are needed to delineate the role of oxidative stress in CS and cardiometabolic shock.

### Occult infection

Infection may lead to further inflammation and worsen the degree of shock, but early identification of infection in patients with cardiometabolic shock may be difficult. The clinical picture, laboratory values, and hemodynamic profile of patients with cardiometabolic shock share similarities with septic shock, including fever, leukocytosis with neutrophil predominance, and vasodilation with low SVR. Inflammatory biomarkers to support early suspicion of sepsis, such as CRP and procalcitonin, may lack clinical utility and further confound the diagnosis as patients with CS have similar peak values with or without confirmed concomitant infection (75). Patients with CS are subject to multiple complications and undergo interventions that increase the risk of developing concomitant infection, such as pulmonary congestion, cardiac arrest requiring cardiopulmonary resuscitation, acute respiratory failure requiring mechanical ventilation, invasive access site and indwelling urinary catheter insertion. Clinical signs of SIRS may be present in as high as 53.8% of patients with CS, likely higher in patients with cardiometabolic shock (75). In the SHOCK trial, culture positive sepsis was reported in 13.3% of patients (4, 76), and they represented the majority of SIRS-positive patients (74%); however, other studies have shown much lower rates of culture positivity, with only 4.1% of CS patients with SIRS having positive blood cultures (77). In a prospective observational study of 80 patients with CS, 37 (46.3%) were found to have infection (75). The median time to onset of infection was 48 h, with respiratory tract being the most common source of infection.

Not only is it difficult to determine if early stages of concomitant infection may be present, but the poor perfusion of cardiometabolic shock may contribute to the elevated risk of infection. Lower SVR in cardiometabolic shock has been associated with greater likelihood of culture-positive sepsis (4). Poor perfusion may lead to thinning of intestinal mucosa increasing intestinal permeability and translocation of bacterial endotoxin from intestinal flora, further exacerbating hypotension (78–80). Likewise, sepsis may further exacerbate cardiac dysfunction and lead to worse outcomes. In septic shock, the RV has been noted to have increasing levels of dysfunction associated with higher mortality (81–83). While fluid resuscitation is a key aspect of overall management of patients with septic shock, it is likely to worsen cardiometabolic shock due to poor pump function, with greater preload further exacerbating myocyte dysfunction. Thus, although differentiating occult or frank infection in the presence of cardiometabolic shock can be confounding, it is a critical consideration in management of patients with CS.

The most critically ill patients being supported with VA-ECLS have been shown to have an increased risk of infection. Two thirds of patients supported by VA-ECLS develop a nosocomial infection, which may result in delayed cardiac transplantation or ventricular assist device implantation, facing increased risk of mortality (84, 85). In comparison to patients in CS treated with medical therapy, initiation of VA-ECLS has been associated with immune system alterations, including increased immature circulating neutrophils, decreased C5a receptor expression, increased expansion of myeloid suppressive cells, T cell dysfunction, and increased pro-inflammatory cytokines including IL-6, IL-8, TNF-α, and anti-inflammatory cytokine IL-10 (86). Such changes in immune function result in immunosuppression and may contribute to the high rate of nosocomial infections in this already complex patient population. Additionally, this may help explain the refractory nature of cardiometabolic shock to mechanical support. It is unclear whether such alterations to the immune system are present with other forms of temporary mechanical support, and whether it would be of clinical relevance. Larger studies with a multimodal approach are needed to expand current understanding of the relationship between bacteremia, circulating endotoxin, and long-term outcomes in patients with CS.

### Right heart failure

The decreased cardiac output in CS often leads to cardiovascular pulmonary congestion and subsequent elevation in right heart pressures. Such elevations in pressure and congestion worsen renal and hepatic injury, further exacerbate acidosis and inflammation, and contribute to the vicious cycle underlying cardiometabolic shock. Among patients with CS receiving mechanical circulatory support, right ventricular congestion correlated with greater risk of mortality (87). Cardiometabolic shock more prominently exhibits right ventricular congestion and elevated right atrial pressures (RAP) > 15 mmHg, compared to other phenotypes of CS (14, 88). The mechanism behind the propensity for development of right heart failure in cardiometabolic shock is multifactorial. RV dysfunction may be induced by elevated pulmonary pressures, metabolic derangements, and acidosis (43, 44). Elevated PCWP and pulmonary artery (PA) pressures are not the sole contributors to RV failure in cardiometabolic shock as the PCWP and PA pressures were not significantly higher compared to other CS phenotypes without associated RV failure (14). As described above, lactic acidosis, pro-inflammatory state, and ROS may independently contribute to the subsequent RV failure associated with cardiometabolic shock.

Supporting patients with biventricular failure is expectedly more complex than patients solely with LV dysfunction. The presence of biventricular dysfunction is likely to contribute to the increased mortality in cardiometabolic shock (83). Regarding mechanical support, VA-ECLS is commonly utilized for patients with biventricular failure allowing for interventions and possible recovery (89). The use of VA-ECLS may be limited by several factors, including patient characteristics and comorbid conditions, vascular access, left ventricular dysfunction without a sufficient left ventricular venting strategy, and its elevated risk of complications. CS patients at risk of developing right heart or biventricular failure, such as those with cardiometabolic shock, may benefit from early pulmonary artery catheter placement, to provide continuous hemodynamic data and surveillance to prevent further deterioration, as well as provide a tailored approach to mechanical and pharmacologic support (90).

### Hepatic injury

Hepatic injury in CS can be secondary to congestive hepatopathy and decreased end-organ perfusion from decreased cardiac output (91, 92). Hypoxic hepatitis and resultant acute liver failure from CS can lead to passive congestion, resulting in further hepatic dysfunction as well as renal failure. The presence of hepatic injury as part of multiorgan failure is an independent predictor of mortality (91, 93). Hepatic dysfunction predicts worsening CS and an increased need for the use of mechanical and inotropic support, as well as higher mortality as synthetic function of the liver drastically decreases (88, 94, 95). Patients in CS with hypoxic hepatitis have been shown to have 2.5 times higher mortality (96). RV failure commonly seen in cardiometabolic shock worsens congestive hepatopathy, reflected by transaminitis and coagulopathy. Oxidative stress from ischemic hepatitis and reperfusion injury further worsens hepatic function, and this added metabolic component of CS portends poor prognosis (92).

As stated above, hepatic dysfunction has been shown to reduce lactate clearance and prolong lactate normalization in septic shock, but the role of hepatic dysfunction in lactate clearance has not been adequately studied in cardiometabolic shock (97). Early hepatic dysfunction has been associated with higher absolute lactate levels (98). Additionally, acute liver failure can lead to worsening hypotension from splanchnic vasodilation, associated with development of cardiometabolic shock (95, 99, 100). To what degree the metabolic and coagulopathic abnormalities of hepatic dysfunction contribute to the distinct phenotype and increased mortality of cardiometabolic shock is unclear.

### Acute renal injury

As with hepatic injury, AKI is seen in all forms of shock, including CS, primarily due to type 1 cardiorenal syndrome. In CS, decreased renal afferent flow leads to subsequent activation of RAAS to increase volume retention and blood pressure via increasing preload (101). This in turn worsens CS further, as poor pump function makes increased preload maladaptive, often resulting in severe heart failure and impending hemodynamic collapse (101). Studies have demonstrated worsening of SCAI CS stage to be associated with worse renal and hepatic function as above, which may be worsened by metabolic dysfunction (102). While the cardiorenal phenotype of CS is seen with severely reduced glomerular filtration rate (GFR), cardiometabolic shock is associated with more moderately decreased renal function (14, 103). Non-hemodynamic causes of cardiorenal syndrome have been shown to be due to inflammation, sympathetic nervous system overactivation, and effect of cytokines such as TNF-α, IL-1, and IL-6 (101). These additional components may play a role in the progression of cardiometabolic shock and subsequent worsening of renal and cardiac function. Chronic renal replacement therapy has been shown to be beneficial in cardiorenal syndrome (104). Evidence is also emerging that the early use of continuous renal replacement therapy (CRRT) may improve mortality in cardiogenic shock with AKI, as shown in post-operative population (105). Further research is needed to confirm and expand the benefit of CRRT in initial stages of cardiogenic shock prior to the onset of metabolic derangements that may lead to cardiometabolic shock.

### Future directions

Cardiometabolic shock represents a more severe type of CS, marked by treatment-resistant cardiovascular deterioration, maladaptive hemodynamic profile, profound lactic acidosis, systemic and vascular inflammation, and multi-organ dysfunction. It has a high mortality rate, and interventions must include both pharmacologic and mechanical support to counter the complex underlying pathophysiology. The prominent features of lactic acidosis and inflammation may in fact serve as possible targets for additional therapeutics for cardiometabolic shock (33). Further characterization of CS with biomarkers and proteomics may allow for an early identification of those at risk for developing cardiometabolic shock prior to decompensation. Specific molecular inhibitors of inflammation and vasodilation may also be a potential future target. Redefining CS as a spectrum of disease that includes the cardiometabolic phenotype, and addressing the underlying pathophysiology allows for a more targeted approach to treatment for CS and may impact prognosis. Currently, many studies of CS are conducted using the SCAI staging designation and do not separate CS phenotypes in data collection or analysis. This limits data validity and applicability in studying and treating individual CS subtypes. The recent retrospective study by Zweck et al. demonstrates a machine learning algorithm identifying phenotypes that correlates with expert SCAI classification (106). Such promising further directions further stress the importance of dedicated prospective studies with delineated CS subtypes are needed to shed light on cardiometabolic shock. Additionally, larger studies dedicated to comparing CS with SIRS becoming culture positive compared to culture negative CS would be beneficial to differentiate CS with sepsis from cardiometabolic shock.

Proteomics also brings the discussion of further treatment options against various protein complexes with potential to reduce poor outcomes in cardiometabolic shock, with the use of artificial intelligence (AI) to determine which proteomes are more likely to have deleterious effects in CS pathophysiology. In one such example, a protein-based CS patient classifier, CS4P, was created for mortality risk assessment, using a large prospective European registry of patients with CS compared to IABP-SHOCK II trials (107). It includes 2,654 proteins identified by spectrometry proteomics, which are further analyzed using enzyme-linked immunosorbent assay (ELISA) and patient database for correlation with outcomes. Several protein complexes have been implicated in poor mortality in CS, including liver-type fatty acid-binding protein (L-FABP), fructose-bisphosphonate aldolase B (ALDOB), *β*-2 microglobulin (B2MG), and SerpinG1 protein (IC1). L-FABP is a cytosolic protein that participates in fatty acid transport to mitochondria and up-regulated in the setting of cellular damage. ALDOB is an enzyme involved in glucose metabolism in the liver and kidneys and upregulated in multisystem organ failure. B2MG is a protein expressed in all nucleated cells and involved in immune recognition with antigen presentation and is elevated in coronary artery disease (CAD) and atherosclerosis (108). IC1 is a protein involved with inhibition of the complement system, and has been shown to be cardioprotective after myocardial damage, and is inversely related to mortality in CS (109). In fact, IC1 has been investigated as a possible therapeutic target in patients with acute ST elevation MI to reduce reperfusion injury (107). Separate studies have included dipeptidyl peptidase 3 (DPP3), which modulates cardiac contractility (110). DPP3 has been shown to be elevated in patients in refractory CS, including lower cardiac index, lower renal function, and higher severity of CS (111, 112). Other studies have shown DPP3 to be an early predictor of outcome, with early clearance associated with improved outcomes (113). In this translational study, the hazard ratio of early mortality was 1.4 for mice with CS with poor clearance of DPP3. Recent studies have demonstrated a unique proteomic profile in patients with CS compared with patients with heart failure without CS (114, 115).

As seen with the identification and characterization of the CS phenotypes by Zweck et al., machine learning and AI will continue to provide further understanding and assist in the treatment of cardiogenic shock. As stated earlier, subsequent retrospective analysis using machine learning to classify cardiogenic shock phenotypes demonstrate consistency with the CSWG registry. AI allows mass data interpretation and analyses and can also help predict effects of peptide sequences in the field of advancing structural proteomics (116). Machine learning can also be used to interpret clinical and hemodynamic data to predict patient outcomes more effectively (117). In dynamic clinical settings involving critically ill patients, this type of tool will help tailor treatment strategies for patients with CS who may deteriorate to cardiometabolic shock with distinct pathophysiology. While AI can help us further understand disease processes as well as find novel therapeutics, it is currently limited by a need for a large set of data for accuracy and difficulty with widespread access.

### Therapeutic targets

**Figure 2:**
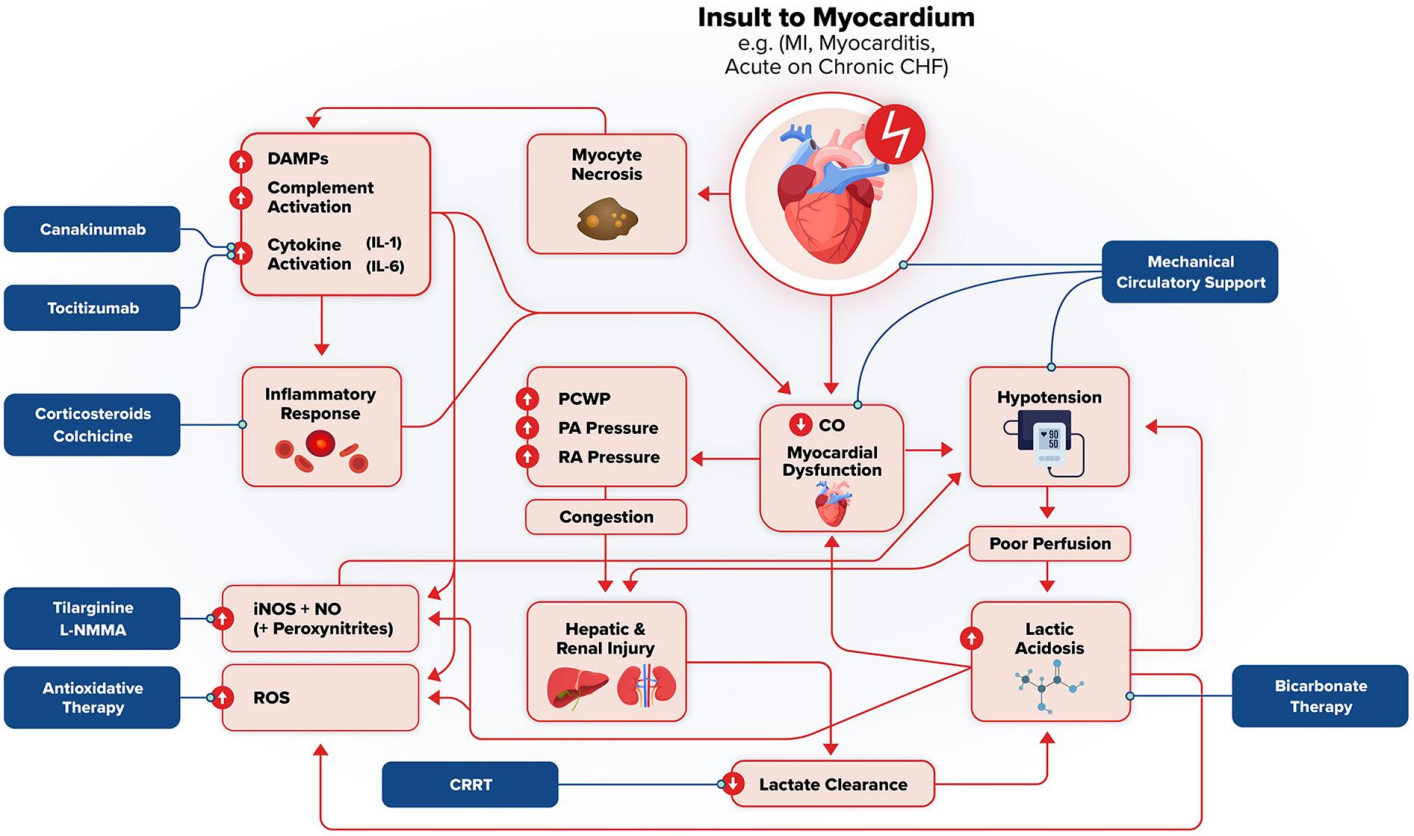
Pathophysiologic framework and potential therapeutic targets in cardiometabolic shock. Acute insult to the myocardium (e.g., MI, myocarditis, acute heart failure, etc.) triggers myocyte necrosis, systemic inflammation, and subsequent hemodynamic collapse. Damage associated molecular patterns (DAMPs), complement activation, and pro-inflammatory cytokines drive this systemic inflammation, which act to further worsen myocardial dysfunction and end-organ damage. This worsening cardiac output leads to hypotension, poor perfusion, lactic acidosis, and ischemia/reperfusion injury which reflect and exacerbate shock. Reactive oxygen species (ROS, inducible nitric oxide synthase (iNOS)-mediated vasodilation, and hepatic/renal injury reinforce the downward spiral through worsening metabolic and lactic acidosis, impaired lactate clearance, and metabolic derangement. Increased intracardiac pressure from myocardial dysfunction and subsequent systemic congestion further compromise end-organ function. Potential therapeutic interventions are highlighted, targeting inflammation (canakinumab, tocilizumab, corticosteroids, colchicine), oxidative stress (antioxidant therapy), nitric oxide signaling (tilarginine, L-NMMA), metabolic acidosis (Bicarbonate therapy, renal replacement therapy, and hemodynamic instability (mechanical circulator support). These strategies aim to interrupt the downward spiral of cardiometabolic shock and mitigate multiorgan failure.

The unique pathophysiology of cardiometabolic shock allows possible therapeutic targets against its various components. Figure 2 illustrates a general framework of the development of cardiometabolic shock and potential therapeutics targeting specific aspects of its pathophysiology. Table 2 summarizes pathophysiology and potential therapeutic targets. Tachycardia and profound hypotension with congestion can be addressed with pharmacologic vasopressors (e.g., norepinephrine) or inotropes such as dobutamine or milrinone and mechanical circulatory support. The DanGer Shock trial demonstrated that use of a microaxial flow pump in patients with cardiogenic shock from myocardial infarction (ST segment elevated) had improved mortality from all causes than standard care alone (118). However, the ECLS-Shock Trial did not demonstrate improved mortality with use of ECLS in patients with infarct-related CS who underwent early revascularization compared with patients without ECLS (119). Additionally, the use of vasopressors and inotropes have lacked evidence to suggest they improve outcomes (120). Profound hypotension is largely caused by massive vasodilation caused by cytokine and inflammatory dysregulation. Several targets may include IL-6, IL-1B actions, and nonspecific anti-inflammatory medications such as steroids or colchicine.

**Table 2:** Therapeutic strategies in cardiometabolic shock: A comparison of pathophysiology, therapeutic targets, mechanisms, and outcomes.

Pathophysiology component	Therapeutic examples	Mechanism of therapy	Reported outcomes	Key references
Profound Hypotension/Low Cardiac Output	Vasopressors (norepinephrine), inotropes (dobutamine, milrinone); mechanical circulatory support (VA-ECMO, Impella)	↑Vasoconstriction, ↑contractility, hemodynamic support	Hemodynamic improvement; no proven mortality benefit; DanGer trial showed reduced mortality with Impella; ECMO trial (ECLS-Shock) trial without improved mortality	(118–120)
Systemic Inflammation	Tocilizumab (IL-6 inhibitor), Canakinumab (IL-1β inhibitor), corticosteroids, colchicine	Cytokine inhibition; anti-inflammatory	Reduced inflammation post-MI; mixed overall cardiovascular outcomes, not studied in cardiometabolic shock specifically	(121–126)
Nitric Oxide Dysregulation	L-NMMA (selective NOS inhibitor), Tilarginine (nonselective NOS inhibitor	Inhibition of NO production: ↑ vascular tone	Improved BP hemodynamics but no mortality benefit for Tilarginine, L-NMMA showed improved MAP and UOP within 24 h	(127)
Metabolic/Lactic Acidosis	IV bicarbonate, renal replacement therapy	Buffer acidosis, improve lactate clearance and improve response to vasopressor therapy	Improved survival in severe acidosis using bicarbonate; renal replacement therapy may improve acidosis but there is no clear mortality benefit in cardiogenic shock	(36, 45–47)
Oxidative Stress/ROS	Antioxidants including *α*-lipoic acid, N-acetylcysteine, flavonoids, delivered nanoparticles, mitochondrial transplantation	Scavenge ROS, reduce oxidative damage	Early data for mitochondrial transplantation suggests clinical benefit in pilot studies, antioxidants show early/preclinical evidence of improved myocardial function	(74, 128)
Multiorgan Failure	Early renal replacement therapy, decongestion with diuretics and renal replacement, supportive care	Reduce myocardial demand and congestive organ damage	Improved outcomes noted in select populations, limited evidence in cardiometabolic shock	(91, 93, 95, 105)

Tocilizumab is a monoclonal antibody targeting IL-6 and used in a variety of systemic inflammatory states, including cytokine storm (121). Given acute MI is followed by vascular and myocardial inflammation, recent studies have investigated outcomes with anti-inflammatory therapies following MI (122). Further investigation of patients at risk for CS with dobutamine and tocilizumab [Low-Dose Dobutamine and Single-Dose Tocilizumab in Acute Myocardial Infarction with High Risk of Cardiogenic Shock (DOBERMANN Trial)] is targeting IL-6 to mitigate potential inflammatory or neurohormonal effects on hemodynamic instability that may arise after acute MI. The Assessing the Effect of Anti-IL-6 Treatment in Myocardial Infarction (ASSAIL MI) Trial revealed that tocilizumab increased myocardial salvage as seen on magnetic resonance imaging (MRI) 3–7 days post MI compared to control in patients with acute MI (123). Canakinumab, a monoclonal antibody targeting IL-1B, has been studied in the Cantos Trial, in which patients with previous MI with a high CRP were treated with canakinumab, and has been shown to decrease the rate of recurrent cardiovascular events when compared to control groups (124). These agents have yet to be studied specifically in cardiometabolic shock or CS at large.

Low-dose steroid therapy is currently being studied in CS in the Low-Dose Corticosteroid Therapy for Cardiogenic Shock in Adults (COCCA) trial, as steroids demonstrated improved vasopressor sensitivity and improvement of arterial pressure (125). Similarly, colchicine, an anti-inflammatory medication, has been studied after recent MI in the Colchicine Cardiovascular Outcomes Trial (COLCOT), which reported decreased risk of further cardiovascular events in the colchicine group when compared to placebo (126). L-NMMA, a selective NOS inhibitor, was studied in the treatment of CS and showed that arterial pressure increased within 10 min of administration, the increase sustained, and had increased urine output after 24 h of treatment (127). Such therapies may be of greater benefit to patients with CS in which inflammatory and vasodilatory mediators play a significant role in poor outcomes. Early application of such therapies at the onset with early identification of cardiometabolic shock may ameliorate the potential for decompensation. As previously mentioned, hepatic and renal dysfunction may be improved by treating congestion. It would be beneficial to study the use of early renal replacement therapy to improve lactic acid clearance and renal dysfunction as a potential treatment for cardiometabolic shock. This may alleviate the progression of right heart dysfunction.

There has been ongoing investigation into antioxidant therapy, including *α*-lipoic acid, N-acetyl cysteine, flavonoids, quinones, and electrophiles to reduce oxidative stress on vascular and cardiac cells (128). Nanoparticles have been investigated as mechanisms for delivery of these antioxidants using liposomes, polymeric micelles, and conjugated polymers. Targets include activated endothelium and atherosclerotic arteries. Nanoparticle technology may open the door for targeted therapy in patients who are undergoing severe cardiac inflammation and oxidative stress. Anti-oxidative treatments may warrant further investigation, given the role of ROS in the cardiometabolic shock profile, and the promise of nanotechnology delivering anti-oxidative molecules to decrease ROS damage to cardiac tissue. Additionally, there are potential proteome targets which have been associated with CS phenotypes. Consideration for CS phenotype should be evaluated in the outcomes of future CS trials at large.

### Summary

This review delves into phenotypes of CS with a focus on cardiometabolic shock and demonstrates the high degree of heterogeneity in its spectrum of disease beyond cardiac hemodynamics. The profound hemodynamic instability of cardiometabolic shock is refractory to pharmacologic and mechanical support, which is largely contributed to by I/R injury characterized by metabolic dysfunction, inflammation, excessive oxidative stress, and lactic acidosis which acts both as a marker of malperfusion as well as a deleterious agent on cardiac myocyte function. This myocyte dysfunction leads to right heart failure out of proportion to left heart failure, which further exacerbates metabolic dysfunction seen in renal and hepatic injury, which both are secondary and contribute to metabolic dysfunction. The cardiometabolic phenotype portends the highest mortality of subtypes of CS, and current practices do not properly identify or treat this subtype. Future and ongoing randomized trials that disregard this heterogeneity in phenotype and severity are likely to result in a null hypothesis, not identifying the patient subtype a specific intervention may benefit. Therefore, further randomized trials that control for CS phenotypes are necessary to investigate diagnostic criteria for cardiometabolic shock and novel therapies, which could potentially lead to improved mortality in these patients. Subcategories of contributing disease process should be investigated as it pertains to definitions or as approaches for therapeutic targets. Potential interventions include anti-inflammatory medications like steroids, nitric oxide synthase inhibitors, and antioxidant therapy including nanoparticles as delivery devices to reactive oxygen species. Some tools (including proteomics, AI, etc.) being investigated have the potential to help differentiate therapeutic targets which can be individualized based on patient disease characteristics and manifestations, and to help identify patients with variant phenotypes.
